# The Establishment of a Three-Chamber Social Preference Assay in Larval Zebrafish for Neurotoxicity Assessment and Neurodevelopmental Gene Phenotyping

**DOI:** 10.3390/biology15211926

**Published:** 2026-10-23

**Authors:** Qiaosen Shen, Huaqing Lan, Shasha Wei, Jiahui Li, Xiaoyan Lin, Shujuan Li, Feng Zhao

**Affiliations:** 1 Fujian Metrology Institute, Fuzhou 350003, China; sqs9208@163.com (Q.S.); 13685025258@163.com (H.L.); wss9770604@163.com (S.W.); 13489951036@163.com (J.L.); linxy4522@gmail.com (X.L.); 13606931196@163.com (S.L.); 2 Experimental Center, Shandong University of Traditional Chinese Medicine, Jinan 250355, China

**Keywords:** larval zebrafish, three-chamber social preference, neurotoxicity, phenotyping

## Abstract

Zebrafish serve as a vertebrate model for assessing environmental neurotoxicity and exploring neurobehavioural phenotypes. However, rapid detection systems for larval zebrafish social preference are still lacking, which restricts efficient behavioural phenotyping and toxicological screening. Here, we developed and validated a 3D-printed three-chamber social preference assay for larval zebrafish that is fully compatible with the Noldus behavioural analysis platform. We confirmed that 14 days post fertilisation (dpf) zebrafish larvae display social preference toward conspecifics, whereas 7 dpf larvae have no significant social bias, defining the key developmental time window for larval social preference. The utility of this method was validated using multiple chemical and genetic models. Acute ethanol and developmental bisphenol A (BPA) exposure significantly disrupted larval social preference with altered locomotor activity, while equal-dose bisphenol AF (BPAF) exposure only impaired locomotion without affecting sociality, indicating distinct neurotoxic effects of BPA analogues. Additionally, two models with neurodevelopmental gene knockdown showed distinct alterations in larval social preference when assessed using this assay. Collectively, this study establishes an efficient larval social preference assay in zebrafish. It provides a feasible methodological tool for environmental neurotoxicity evaluation and phenotypic analysis of neurobehavioural phenotypes.

## Simple Summary

Zebrafish larvae serve as valuable model organisms for neurotoxicity and neurodevelopmental research, yet standardised tools to measure larval social preference are still lacking, limiting toxicant screening and genetic phenotypic evaluation. Here, we developed and validated a 3D-printed three-chamber social preference assay for larval zebrafish, which is fully compatible with the mainstream behavioural tracking system. We found that zebrafish larvae display social preference at 14 days post-fertilisation (dpf) but show no significant social bias at 7 dpf, identifying the critical developmental window for larval social preference. We validated this assay using chemical exposure and morpholino-mediated gene-knockdown models. Acute ethanol and developmental bisphenol A (BPA) exposure impaired social preference and altered locomotor activity. At the same concentration, bisphenol AF (BPAF) only disturbed swimming performance without affecting larval social preference, revealing different neurotoxic effects between these two bisphenol analogues. Furthermore, this assay was capable of detecting social preference deficits in certain gene-knockdown larval models. Compared with adult-fish based protocols, this larval-focused assay reduces time and experimental costs. It provides a practical tool for environmental neurotoxicity assessment and neurobehavioural phenotypes in zebrafish.

## 1. Introduction

Owing to their high fecundity, rapid external embryonic development, and neural circuit regulatory mechanisms highly conserved with mammals, zebrafish serve as a complementary vertebrate model for preliminary neurotoxicity screening and mechanistic exploration rather than a replacement for mammalian models. Social behaviour is indispensable for the survival and reproduction of zebrafish [1,2,3]. AB changes in social behaviour serve as one of the readouts of neurobehavioural dysfunction [4,5,6,7,8]. Therefore, establishing a rapid and quantifiable social behavioural assay in zebrafish is of great significance for evaluating the neurotoxicity of environmental pollutants and investigating neurobehavioural phenotype.

The three-chamber social test is a well-established and widely used paradigm for assessing social preference in zebrafish. Most studies have focused on adult zebrafish, using a camera to record the duration and entry frequency of experimental fish in the social area adjacent to conspecifics. These indicators are applied to assess social preference, and the paradigm has been widely utilised for neurotoxicity screening of environmental pollutants and exploring neurobehavioural phenotypes [9,10,11,12,13,14,15]. However, the long rearing cycle and high maintenance costs associated with adult zebrafish impede rapid screening of pollutant neurotoxicity and genetic phenotypic profiling. In addition, the critical developmental time window of larval social preference in zebrafish and the applicability of corresponding detection methods remain poorly defined. Meanwhile, although larval behavioural analysis platforms such as Noldus have been widely adopted, dedicated three-chamber social preference test apparatus and standardised detection protocols for larval zebrafish that are compatible with such platforms are still lacking [16,17].

In this study, we developed a three-chamber social preference detection device and a corresponding protocol for larval zebrafish. The apparatus and method are compatible with the mainstream behavioural analysis system (Such as such as Noldus systems) for larval zebrafish. Furthermore, neurotoxicant (Bisphenol A and ethanol) exposure and models for neurodevelopmental gene (*micall2b* and *lphn3.1*) knockdown were used to validate the efficiency of the established method, providing a methodological tool for neurotoxicity screening of environmental pollutants and phenotypic analysis of neurobehavioural phenotypes.

## 2. Materials and Methods

### 2.1. Fish Husbandry

Wild-type (WT) AB line zebrafish (Catalog ID: CZ1) were originally obtained from the China Zebrafish Resource Center (CZRC), Wuhan, China and subsequently maintained in the fish facility of Shandong University of Traditional Chinese Medicine. All zebrafish experiments were performed in accordance with the ARRIVE guidelines and approved by the Institutional Animal Care and Use Committee of Shandong University of Traditional Chinese Medicine (SDUTCM20260303606). Adult AB line zebrafish were cultured in 3 L tanks with recirculating water maintained at 28 °C under a 14 h light/10 h dark photoperiod. Zebrafish embryos were obtained from naturally spawning adult mating pairs and incubated in a thermostatic incubator.

### 2.2. Chemical Exposure Paradigm

Ethanol (CAS 64-17-5), bisphenol A (BPA, CAS 80-05-7), and bisphenol AF (BPAF, CAS 1478-61-1) were obtained from Sigma-Aldrich, St. Louis, MO, USA. Separate 43 mM stock solutions of BPA and BPAF were prepared in pure dimethyl sulfoxide (DMSO, CAS 67-68-5) and stored at −20 °C. Zebrafish embryos were exposed to 0.43 μM BPA, 0.43 μM BPAF, or vehicle control (0.1% DMSO) from 2 hpf to 120 hpf in petri dishes, with 50 embryos allocated per dish. At 5 dpf, all exposed embryos were rinsed three times with embryo medium (EM). At 5 dpf, all exposed embryos were rinsed three times with embryo medium (EM), transferred to rearing tanks (approximately 50 larvae per tank), and maintained until 14 dpf.

For acute ethanol exposure, individual 14 dpf wild-type zebrafish were placed in mesh strainer inserts (one larva per insert). Each insert was submerged in a single well of a standard 6-well plate preloaded with 15 mL fish water containing 1% ethanol. Fish were subjected to ethanol treatment for 20 min; larvae incubated in clean fish water served as the vehicle control group. Upon completion of exposure, all mesh inserts were transferred to a separate 6-well plate filled with fresh fish water for a 15 s rinse. Zebrafish were immediately transferred to the three-chamber social preference apparatus after washing for subsequent behavioural assessment. All chemical exposure experiments were performed with four independent tanks (biological replicates). Each replicate was maintained in a separate tank, with approximately 50 larvae per tank.

### 2.3. The Three-Chamber Social Preference Apparatus and Test Procedure

**Figure 1:**
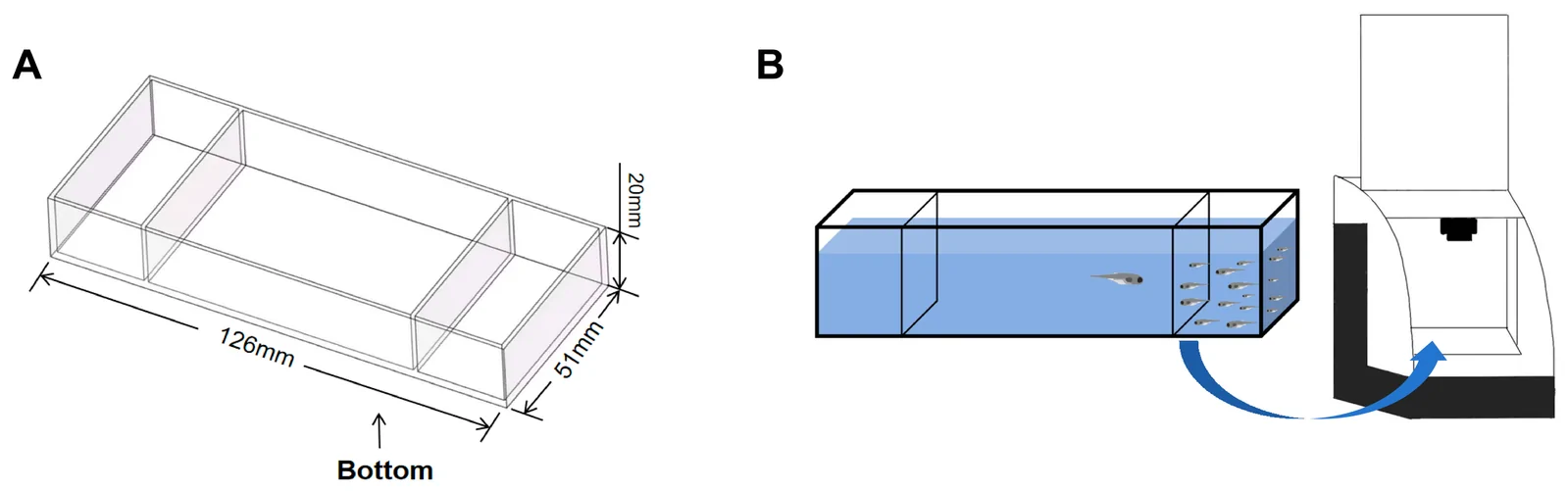
The schematic diagram of the three-chamber social preference apparatus for zebrafish. (**A**) Overall dimensions of the transparent testing apparatus: total length, 126 mm, width, 51 mm, height, 20 mm, divided into three independent compartments by two transparent partitions. (**B**) An illustration of the experimental setup for 3-chamber social interaction test. Subject zebrafish were placed within the central testing chamber, and one side compartment was designated as the stimulus chamber containing age-matched conspecific fish. All behaviours test was captured via the Noldus zebrafish behaviour analysis platform.

In this study, a three-chamber social apparatus was developed and fabricated via 3D printing using fully cured transparent UV resin, with overall dimensions of 126 mm (length) × 51 mm (width) × 20 mm (height) (Figure 1A). This custom 3D-printed apparatus was developed solely for non-commercial academic research. Prior to the test, 50 mL of zebrafish fish water was added into the central test chamber before the subject fish were placed inside. The left and right side chambers were each filled with 18 mL of fish water: one chamber contained 24 age-matched conspecific zebrafish serving as the social stimulus chamber, while the other chamber remained empty of fish as the blank control chamber.

**Figure 2:**
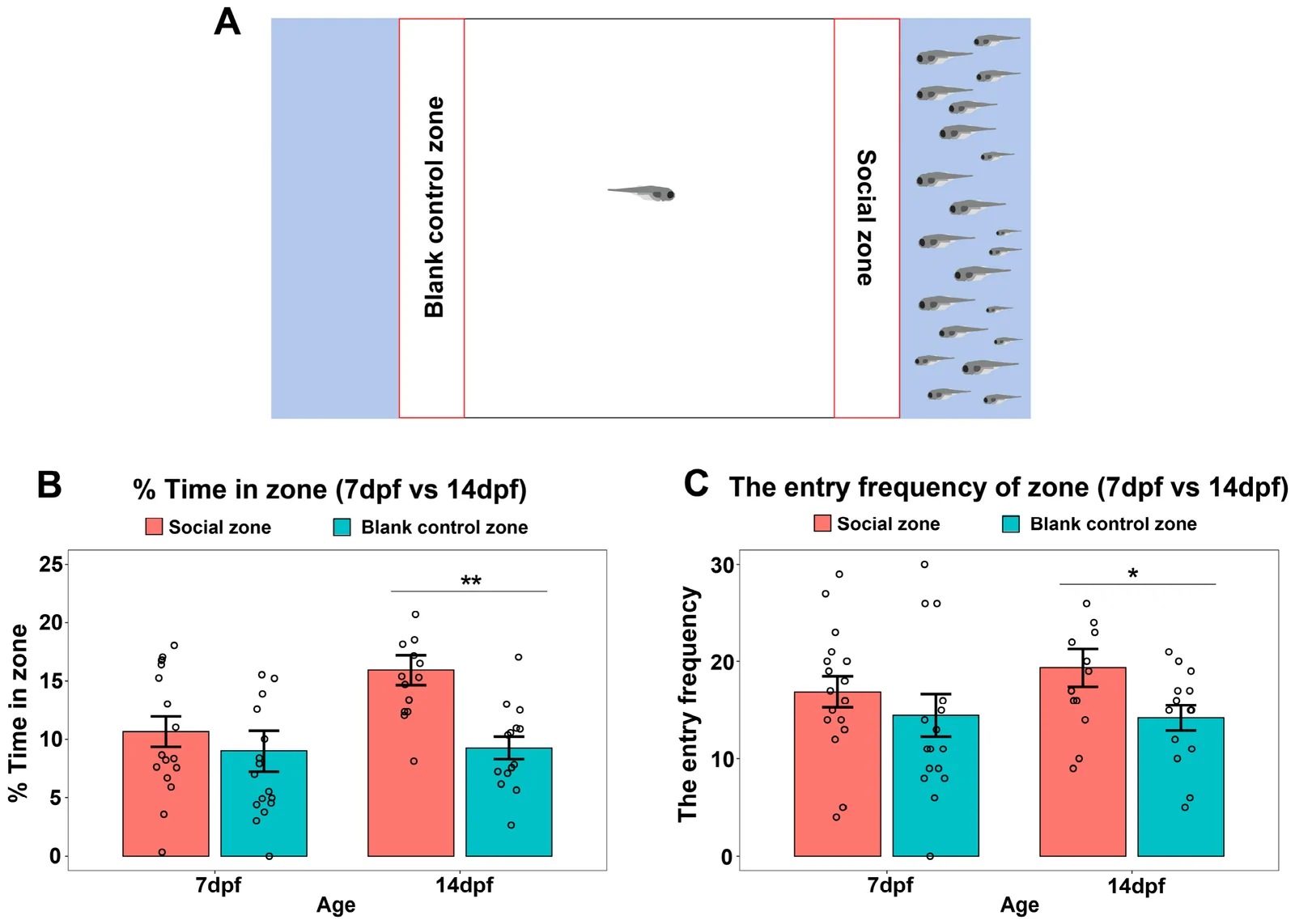
Zebrafish three-chamber social preference assay and behavioural analysis of 7dpf and 14dpf zebrafish. (**A**) The top view of the three-chamber social preference test tank. The right red rectangular outline defines the social zone with adjacent to age-matched conspecific stimulus fish, while the left red rectangular outline marks the blank control zone with no conspecific fish. (**B**) The average time staying in social zone (%) and blank control zone of the 7dpf and 14dpf zebrafish, respectively (7 dpf: n = 3 independent tanks per group; 5–6 larvae subsampled from each tank; paired *t*-test, *p*-value = 0.5321. 14 dpf: n = 3 independent tanks per group; 4–5 larvae subsampled from each tank; paired *t*-test, *p*-value = 0.001153). (**C**) The average entry frequency of the social zone a blank control zone of the 7dpf and 14dpf zebrafish (7 dpf: n = 3 independent tanks per group; 5–6 larvae subsampled from each tank; paired *t*-test, *p*-value = 0.382. 14 dpf: n = 3 independent tanks per group; 4–5 larvae subsampled from each tank; paired *t*-test, *p*-value = 0.026). All values are presented as the mean of tank-averaged values ± SEM. Overlaid dots denote data from individual larvae. * and ** stand for *p*-value < 0.05 and *p*-value < 0.01, respectively.

Previous studies have demonstrated that the social zone for zebrafish is generally defined within a region of 1.5–2 body lengths from the edge of the conspecific stimulus chamber [8,18]. Accordingly, a 51 mm × 12 mm social zone was delineated adjacent to the edge of the conspecific stimulus chamber, and a blank control zone of identical dimensions was set beside the blank control chamber (Figure 2A). All behavioural recordings were conducted using the Noldus behavioural tracking system (DanioVision, Noldus Information Technology BV, Wageningen, The Netherlands). Zebrafish embryos were fully mixed and randomly allocated to experimental groups. The experimenter who transferred zebrafish into the test apparatus was blind to treatment groups. The apparatus containing the test fish was placed in the tracking box for a 10 min dark acclimation period, after which social preference was recorded for 15 min under 100 lx illumination. The system automatically tracked and collected data of zebrafish in the central test zone, social zone and blank control zone, and all behavioural data were analyzed using EthoVision XT 17 software. The video analyst remained blind to treatment groups until data sorting was finished.

### 2.4. Morpholino (MO) Microinjection in Zebrafish

The *micall2b*-MO (sequence:5′-AAGTGAATGGTATCTTACCCGTTGA-3′) and *lphn3.1*-MO (sequence: 5′-ATGTGAGTGTATCTCTGTACCTGAA-3′) used in this study have been previously validated for their knockdown specificity and efficiency in published studies [19,20,21]. At the one-cell stage, zebrafish embryos were microinjected with 1 nL of *micall2b*-MO or *lphn3.1*-MO working solution (0.25 μM). Embryos in the control group received an equal volume of standard control-MO (sequence: 5′-ATCTGACTGTATGTCTCTACCTCAA-3′). All injected zebrafish larvae were raised continuously until 14 dpf, after which larval social preference assays were performed. All gene knockdown experiments were performed with four independent tanks (biological replicates). Each replicate was maintained in a separate tank, with approximately 50 larvae per tank.

### 2.5. Statistics

All data were first tested for normality and homogeneity of variance using the Shapiro–Wilk test and Levene’s test, respectively, in RStudio (2024.12.1), to determine whether parametric or non-parametric tests were appropriate. Paired *t*-test or Wilcoxon signed-rank test was applied for comparisons between the social zone and blank-control zone, as measurements were collected from the same individual zebrafish. Differences between two independent groups were compared using Student’s *t*-test or the Mann–Whitney U test. One-way analysis of variance (ANOVA) followed by Tukey’s post hoc test was used for pairwise comparisons among multiple groups. When the normality or homogeneity assumptions were violated, the non-parametric Kruskal–Wallis test followed by Dunn’s post hoc test with Bonferroni correction was used. For intergroup comparisons between independent treatment groups, tank-averaged values were calculated and used for statistical comparisons. Statistical analyses and plotting were performed in RStudio. Statistical significance thresholds were defined as follows: * *p* < 0.05, ** *p* < 0.01, *** *p* < 0.001.

## 3. Results

### 3.1. 14 dpf Zebrafish Exhibit Significant Larval Social Preference

To determine the developmental onset of social preference in larval zebrafish, behavioural assays were performed on zebrafish larvae at 7 and 14 dpf. At 7 dpf, no significant differences were observed between the time spent and frequency of entry in the social zone versus the blank control zone (Figure 2B,C). In contrast, 14 dpf larvae exhibited significantly higher %time in social zone and entry frequency in social zone compared with blank control zone (Figure 2B,C), indicating that social preference is well established by 14 dpf in zebrafish.

### 3.2. Validation of Larval Zebrafish Social Preference Assay with Ethonal and BPA Exposure

BPA and ethanol were selected as positive controls for assay validation. Both are behavioural neurotoxicants, and prior publications have quantified their effects on zebrafish social phenotypes, including the time spent in the social zone and entry frequency. For BPA, multiple studies using Noldus Ethovision have characterised its impacts on social behaviour [10,13,18,22,23,24]. For ethanol, numerous Noldus-based zebrafish behavioural studies have demonstrated that acute ethanol exposure alters social phenotypes [25,26,27,28,29].

**Figure 3:**
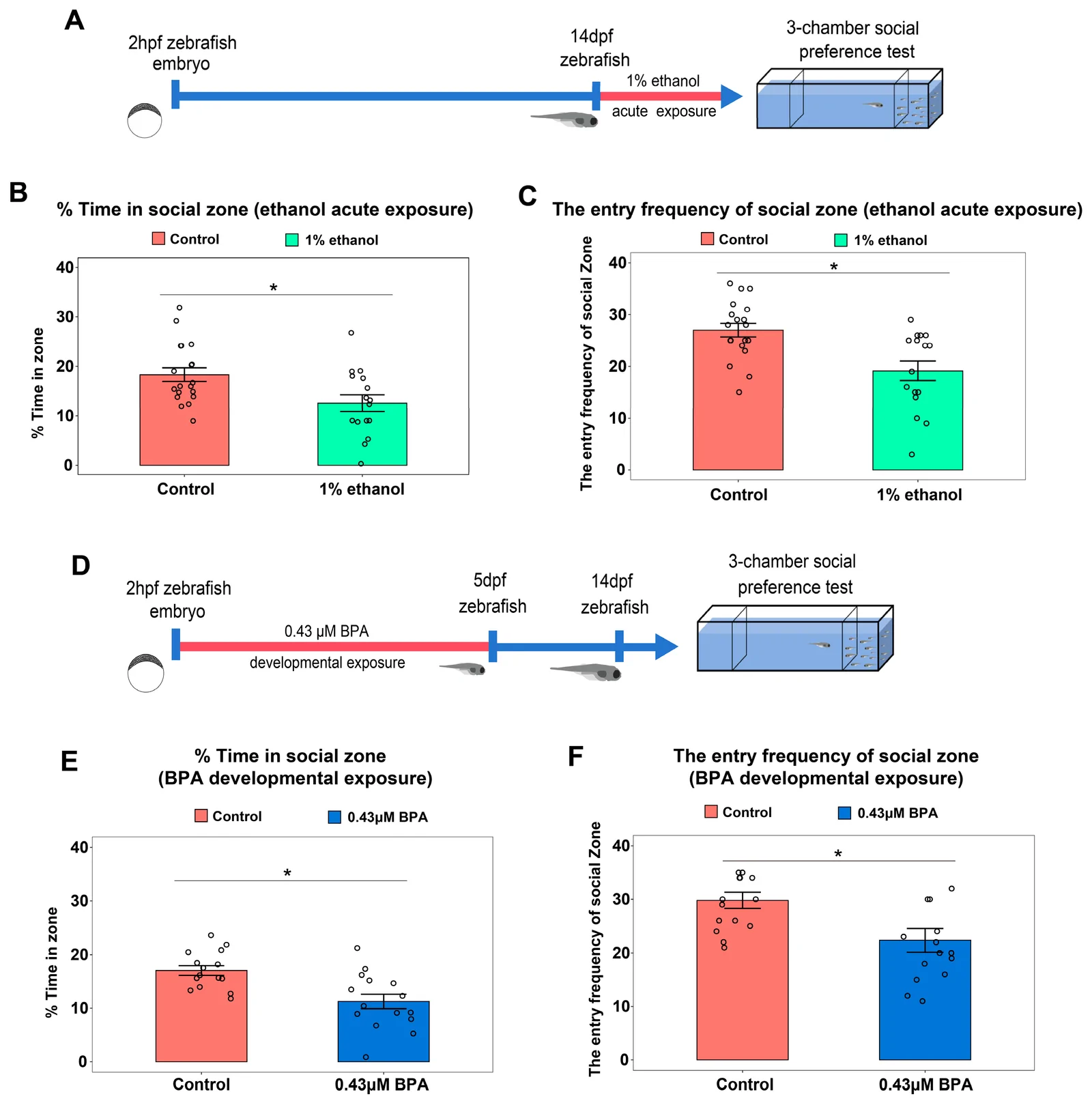
Behavioural validations of zebrafish three-chamber social preference test following ethanol and BPA treatment. (**A**) The schematic timeline of acute ethanol exposure paradigm. WT zebrafish embryos developed normally until 14dpf, followed by short-term 1% ethanol acute administration prior to three-chamber social preference recording. (**B**) The bar plot showing the proportion of total test duration zebrafish occupied the social zone in control and acute 1% ethanol groups (n = 4 independent tanks per group; *t*-test, *p*-value = 0.0286). (**C**) A statistical comparison of social zone entry frequency between control and ethanol-treated zebrafish (n = 4 independent tanks per group; 4–5 larvae subsampled from each tank; *t*-test, *p*-value = 0.0277). (**D**) The schematic timeline of developmental BPA exposure paradigm. Zebrafish embryos were continuously immersed in 0.43 μM BPA solution starting at 2 hpf until 120 hpf (5dpf), then rear in fish water to 14 dpf for social interaction testing. (**E**) The quantification of average %time in social zone in zebrafish with embryonic 0.43 μM BPA developmental exposure versus vehicle control (n = 4 independent tanks per group; 3–4 larvae subsampled from each tank; *t*-test, *p*-value = 0.0263). (**F**) The bar plot illustrating social zone entry frequency in control and developmental BPA-exposed groups (n = 4 independent tanks per group; 3–4 larvae subsampled from each tank; *t*-test, *p*-value = 0.0308). All values are presented as the mean of tank-averaged values ± SEM. Overlaid dots denote data from individual larvae. * stand for *p*-value < 0.05.

Previous studies have shown that exposure to 100 μg/L bisphenol A (BPA) and 1% ethanol can induce alterations in social behaviour and exert neurotoxic effects in zebrafish [18,29,30,31]. Based on these findings, zebrafish were subjected to acute and developmental exposure to ethanol and BPA, respectively. At 14 dpf, zebrafish were subjected to acute exposure to 1% ethanol for 20 min, followed by social preference detection (Figure 3A). Compared with the control group, acute exposure to 1% ethanol significantly reduced the %time spent in the social zone and the entry frequency, accompanied by a notable decrease in swimming distance (Figure S1A). For the BPA treatment group, zebrafish were subjected to developmental exposure from 2 hpf to 120 hpf, and social preference tests were performed when the larvae were raised to 14 dpf (Figure 3D). The results showed that zebrafish with early developmental exposure to BPA exhibited a significant reduction in the %time in the social area and the frequency of entering the social zone (Figure 3E,F), while their swimming distance was significantly increased (Figure S1B). Collectively, these findings confirm that the established social preference apparatus is suitable for conducting behavioural assessment in larval zebrafish.

### 3.3. BPAF Has Different Effects on Laval Social Preference

**Figure 4:**
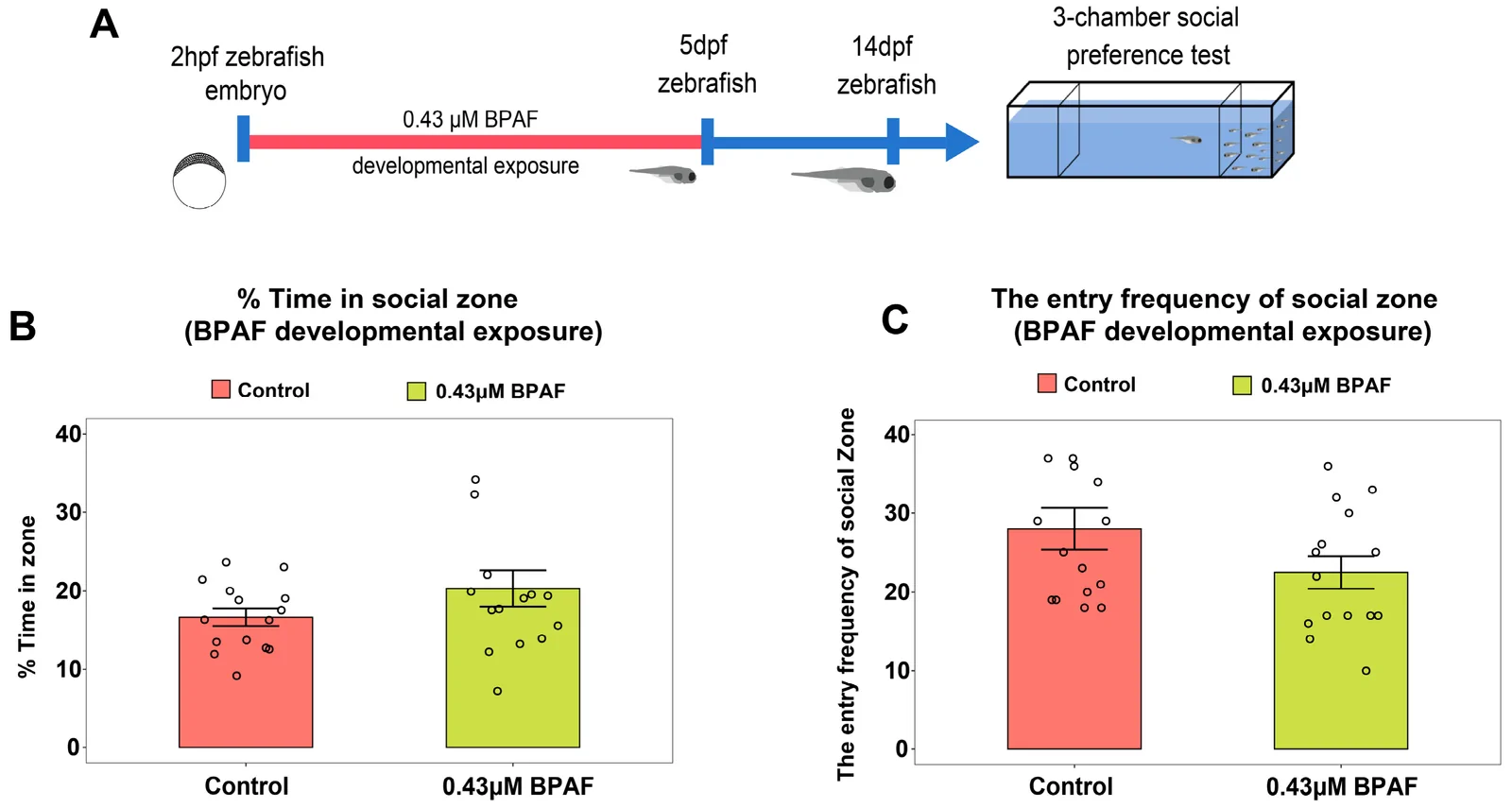
Effects of developmental BPAF exposure on social preference in larval zebrafish. (**A**) The schematic timeline of the developmental BPAF exposure paradigm. Zebrafish embryos were continuously exposed to 0.43 μM BPAF from 2 hpf to 120 hpf (5 dpf), then maintained in fish water until 14 dpf for the three-chamber social preference test. (**B**) The bar plot quantifying the percentage of average %time larvae spent in the social zone in the control and BPAF developmental exposure groups (n = 4 independent tanks per group; 3–4 larvae subsampled from each tank, *t*-test, *p*-value = 0.3837). (**C**) The statistical analysis of social zone entry frequency between the control and BPAF-exposed group (n = 4 independent tanks per group; 3–4 larvae subsampled from each tank; *t*-test, *p*-value = 0.1625). All values are presented as the mean of tank-averaged values ± SEM. Overlaid dots denote data from individual larvae.

BPAF, an industrial alternative to BPA, was selected for comparison since prior work demonstrated it possesses greater toxicity than BPA [22,32,33,34,35]. We hypothesised that BPAF might also induce changes in larval social preference at the same exposure concentration as BPA. In the present study, zebrafish were subjected to developmental exposure to 0.43 μM/L BPAF, a concentration identical to that of BPA in this studies, which did not induce morphological malformations in zebrafish. Larval social preference tests were performed at 14 dpf (Figure 4A). The results showed that compared with the control group, BPAF exposure did not significantly alter the %time spent in the social zone or the frequency of entering the social zone (Figure 4B,C) but significantly reduced the swimming distance of zebrafish (Figure S2), indicating that BPA and BPAF have different neurotoxic effects.

### 3.4. Knockdown micall2b Induced Deficit on Larval Social Preference

Genes *micall2b* and *lphn3.1* are critical genes for neural development in zebrafish. Gene *micall2b* participates in neuronal microtubule development, while *lphn3.1* mediates synaptogenesis and dopaminergic neuron formation. Previous Noldus-based studies have shown their knockdown alters larval locomotor activity [19,20,21,36]. We tested whether these gene perturbations affect larval social preference.

**Figure 5:**
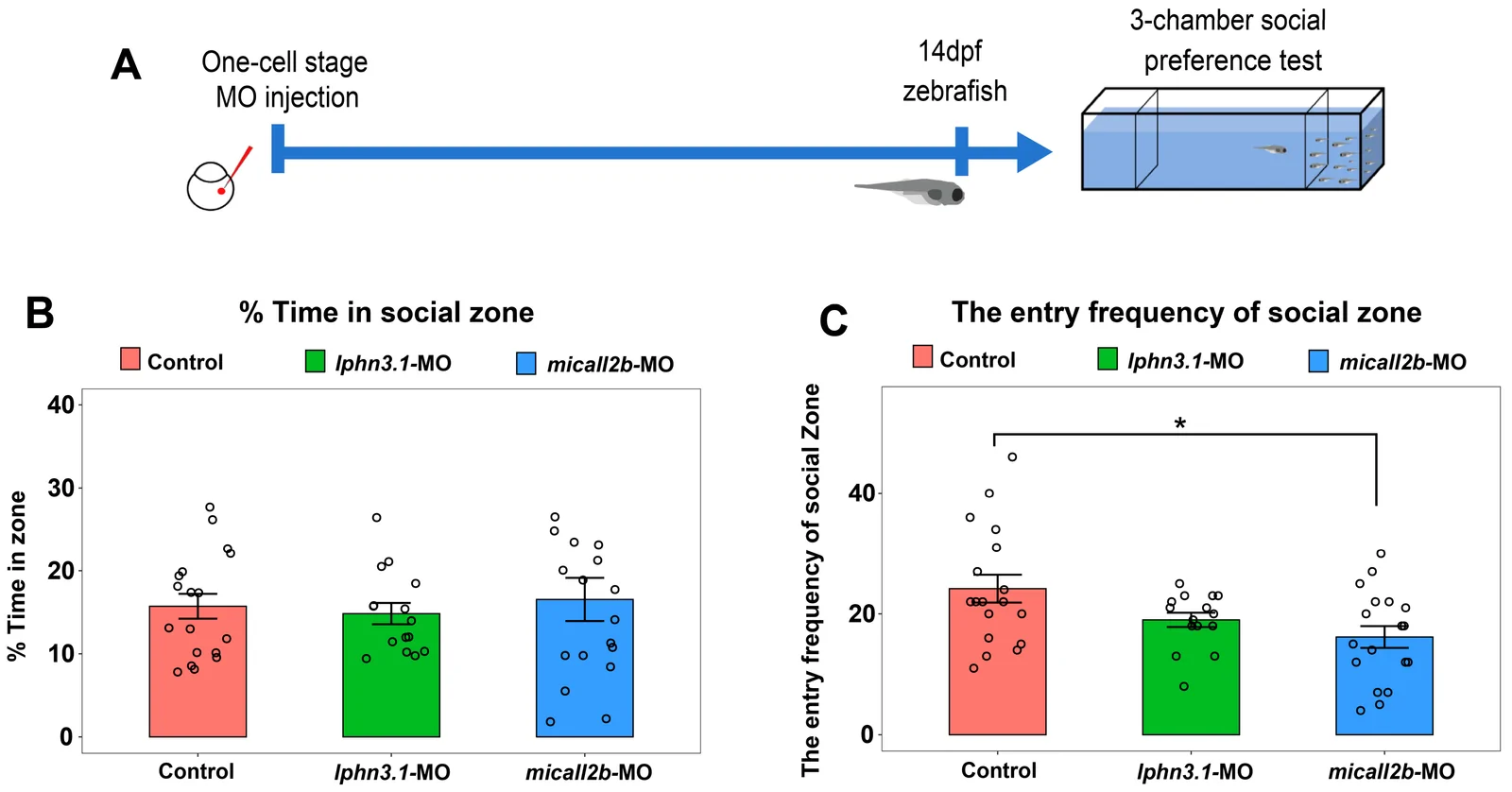
Effects of *lphn3.1* and *micall2b* knockdown on social preference in zebrafish. (**A**) The schematic timeline of morpholino (MO) knockdown assay. MO were microinjected into zebrafish embryos at the one-cell stage, and injected larvae were raised to 14dpf for subsequent three-chamber social preference testing. (**B**) The bar plot showing the %time spent within the social zone across control, *lphn3.1*-MO and *micall2b*-MO groups (n = 4 independent tanks per group; 3–5 larvae subsampled from each tank; one-way ANOVA with Tukey’s post-hoc test, *p*-value = 0.863). (**C**) The quantification of social zone entry frequency in the control, *lphn3.1*-MO and *micall2b*-MO group (n = 4 independent tanks per group; 3–5 larvae subsampled from each tank; one-way ANOVA with Tukey’s post hoc test, *p*-value = 0.0254). All values are presented as the mean of tank-averaged values ± SEM. Overlaid dots denote data from individual larvae. * stand for *p*-value < 0.05.

In this study, one-cell-stage zebrafish embryos were injected with MOs targeting *micall2b* and *lphn3.1*, respectively, and raised to 14 dpf for larval social preference assessment (Figure 5A). The results showed that *micall2b* knockdown significantly reduced the entry frequency into the social zone (Figure 5C), whereas the %time in the social zone and swimming distance remained unchanged (Figure 5B) (Figure S3). In contrast, *lphn3.1* knockdown zebrafish displayed no significant differences from the control group in the %time in the social zone, entry frequency and swimming distance (Figure 5B,C) (Figure S3). These results indicate that the two genes exhibit distinctly different regulatory effects on zebrafish social preference.

## 4. Discussion

Social preference is one of the important behavioural readouts in zebrafish [37,38]. The establishment of behavioural assays is essential for investigating the neurotoxicity of environmental pollutants and characterising neurobehavioural phenotypes. In this study, we developed a three-chamber social preference detection apparatus and matching testing methods applicable to larval zebrafish, which are compatible with the Noldus larval behaviour analysis system (Figure 1A). Through systematic experiments, we clarified the critical developmental time window of social preference in larval zebrafish, verified the reliability of the established methods, and further explored the differential neurotoxic effects of bisphenol analogues as well as the regulatory effects of neurodevelopment-related genes on social preference. This study provides important methodological support for neuroscience and toxicology field.

### 4.1. Zebrafish at 14 dpf Begin to Exhibit Social Preference in the Three-Chamber Apparatus

Previous studies have confirmed that larval zebrafish begin to exhibit conspecific tendency at approximately one week post-fertilisation. These social preference traits are characteristic behavioural features of the zebrafish larval stage [39,40]. In the present study, a three-chamber social preference assay was applied for verification. The results revealed that 7 dpf larvae showed no obvious social bias, with no significant behavioural difference between the social zone and blank control zone, indicating the absence of early social preference at this stage (Figure 2B,C).

In contrast, larval social preference were observed in 14 dpf larvae within the three-chamber system. These larvae spent a significantly higher %time in the social zone (Figure 2B,C) and entry frequency into the social zone was markedly increased, demonstrating that larvae at this stage acquired the capacity to respond to conspecific stimuli and established larval social preference behaviour (Figure 2B,C).

Shoaling, another widely adopted paradigm for evaluating zebrafish social behaviour, generally emerges in larvae at around 28 dpf, representing a relatively later developmental time window [41,42]. Compared with such methods, the three-chamber social preference enables earlier detection of early social alterations, making it more suitable for exploring the underlying mechanisms of early social behaviours modulated by neural development in zebrafish.

### 4.2. Validation of Larval Zebrafish 3-Chamber Social Preference Test by Chemical Exposure

Developmental exposure to BPA disrupts neural circuit construction and induces altered social preference (Figure 3E,F). Acute ethanol exposure directly modulates the function of neural circuit, thereby triggering social behavioural dysfunction (Figure 3B,C). Behavioural alterations resulting from both exposure paradigm can be reliably identified via the three-chamber social preference assay, confirming the applicability of this established detection method.

The results revealed that acute ethanol exposure reduced locomotor activity of zebrafish in the test area (Figure S1A). Evidence has demonstrated that acute ethanol treatment alters the secretion levels of dopamine and serotonin, and the disruption of these two neurotransmitters system directly triggers abnormal motor behaviours [29,43]. In contrast, developmental BPA exposure markedly increased zebrafish locomotion, which was highly consistent with the hyperactive phenotype induced by early BPA exposure [18,44,45]. We found that both BPA and ethanol induced alterations in larval social preference, implying potential shared underlying mechanisms. Accumulating evidence indicates that disrupted GABAergic signalling contributes to impaired zebrafish social behaviour [7,46,47]. Also, our previous work and other reports demonstrated that BPA-triggered behavioural deficits are mainly attributed to the reduction of GABAergic neurons in the zebrafish brain [18,23]. Significant reductions in brain GABA content have been documented in zebrafish displaying ethanol-evoked social behaviour [29]. We speculate that dysfunction of inhibitory GABAergic neurons may be associated with the common mechanism underlying these deficits in larval social preference following BPA and ethanol exposure.

Notably, early developmental exposure to BPAF did not alter the %time spent in the social zone or the frequency of entries into the social zone in zebrafish (Figure 4B,C) yet significantly reduced their total travel distance (Figure S2), which is in contrast to the abnormal social behaviour induced by the same concentrations of BPA exposure. Although previous studies have confirmed that the overall toxicity of BPAF is significantly stronger than that of BPA [32,48,49], the present behavioural results hint at potential behavioural divergence after exposure to the two compounds. Whether these differences arise from disruption of distinct neurotoxic mechanisms requires further experimental validation. These distinct behavioural phenotypes suggest that the three-chamber social preference assay can potentially discriminate the neurotoxicity of different compounds.

### 4.3. Detection of Zebrafish Model of Neurodevelopmental Gene Knockdown

This behavioural detection system can be applied not only to the neurotoxicity screening of environmental pollutants but also to phenotypic analysis of zebrafish models with neurodevelopmental gene knockdown. Both *micall2b* and *lphn3.1* are key genes associated with neurodevelopment in zebrafish. Early knockdown of these genes can alter varying degrees of behavioural and neurodevelopment in zebrafish [19,20,21,36].

In the present study, gene-knockdown zebrafish were reared to 14 dpf and subjected to the three-chamber larval social preference test. Larvae with *micall2b* knockdown exhibited a significant reduction in the frequency of entries into the social zone (Figure 5C), while the %time within this zone remained unchanged (Figure 5B). Whether such changes in larval social preference induced by *micall2b* knockdown are linked to GABAergic neuronal function requires further investigation. In contrast, knockdown of *lphn3.1* produced no significant alterations in larval social preference, suggesting that this gene may not participate in the neural circuits governing larval social preference, and its function is likely primarily related to spontaneous locomotion during early larval development [20,21,50]. Most MO-mediated knockdown models exhibit locomotor abnormalities only at early developmental stages (e.g., 5 dpf). In contrast, the three-chamber social preference assay allows for the detection of long-lasting behavioural deficits induced by gene knockdown.

## 5. Conclusions

This study adopted 3D printing technology to establish and validate a three-chamber social preference assay for larval zebrafish. The present study confirmed that 14 dpf zebrafish larvae display detectable larval social preference in the customised three-chamber apparatus, which is structurally compatible with the mainstream behavioural analysis system. Validation using chemical exposures demonstrated that this method enables us to identify alterations in social preference induced by ethanol and BPA and to discriminate the neurotoxic effects of different compounds. Additional genetic functional tests showed that this approach can detect deficits in social preference caused by MO-mediated gene knockdown. Compared with assays using adult zebrafish, the present method offers improved efficiency and cost-effectiveness. Collectively, these findings support the utility of the established social preference apparatus for behavioural assessment in larval zebrafish. It is suitable for neurotoxicity screening of environmental pollutants and phenotypic evaluation of neurodevelopmental gene knockdown models, providing a practical methodological tool for neurotoxicology and neurobehavioural research.

## Data Availability

The data that support the findings of this study are available upon reasonable request from the corresponding author.

## Supplementary Materials

The following supporting information can be downloaded at https://www.mdpi.com/article/10.3390/biology15211926/s1, Figure S1: Locomotion of 14dpf zebrafish after acute alcohol or developmental BPA exposure; Figure S2: Locomotion of 14dpf zebrafish following developmental BPAF exposure; Figure S3: Locomotion of 14dpf zebrafish after *lphn3.1* and *micall2b* gene knockdown.
