# Supplementary material for: The Establishment of a Three-Chamber Social Preference Assay in Larval Zebrafish for Neurotoxicity Assessment and Neurodevelopmental Gene Phenotyping
Source: Biology (Basel). 2026 Oct 23;15(21):1926. doi: 10.3390/biology15211926 (PMC13761947; doi:10.3390/biology15211926)
Supplement: Supplementary file 1 [file biology-15-01926-s001.zip › biology-4635162-supplementary.pdf]

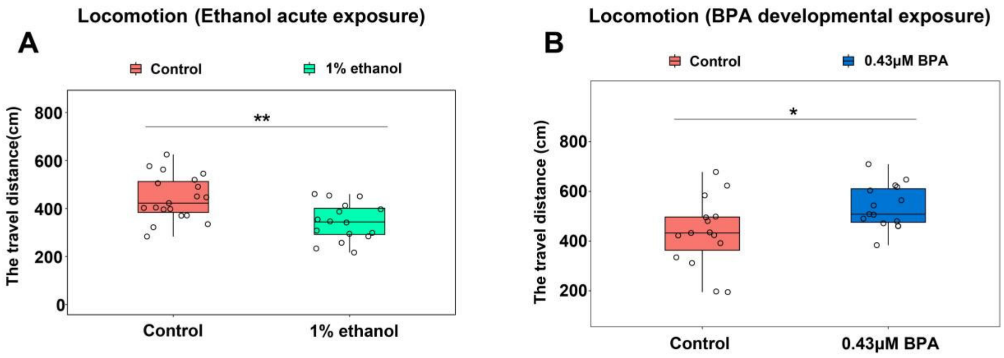

Figure.S1 Locomotion of 14dpf zebrafish after acute alcohol or developmental BPA exposure. (A) Box-and-whisker plot showing average travel distance (cm) in control and 1% acute ethanol-exposed group (n = 4 independent per group; 4-5 larvae subsampled from each tank; *t*-test, *p*-value = 0.0014. (B) Box-and-whisker plot quantifying average travel distance in vehicle control and 0.43 μM developmental BPA-exposed group (n = 4 independent tanks per group; 3-4 larvae subsampled from each tank; *t*-test, *p*-value = 0.0430). Overlaid dots denote data from individual larvae.

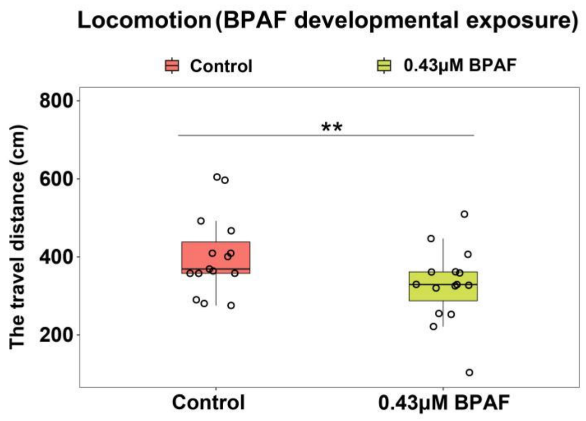

Figure.S2 Locomotion of 14dpf zebrafish following developmental BPAF exposure. Box-and-whisker plot comparing average swimming distance (cm) between vehicle control larvae and zebrafish with developmental exposure to 0.43 μM BPAF (n = 4 independent tanks per group; 3–4 larvae subsampled from each tank; *t*-test, *p*-value = 0.0014). All values are presented as the mean of tank-averaged values ± SEM. Overlaid dots denote data from individual larvae.* and ** stand for *p*-value < 0.05 and *p*-value < 0.01, respectively.

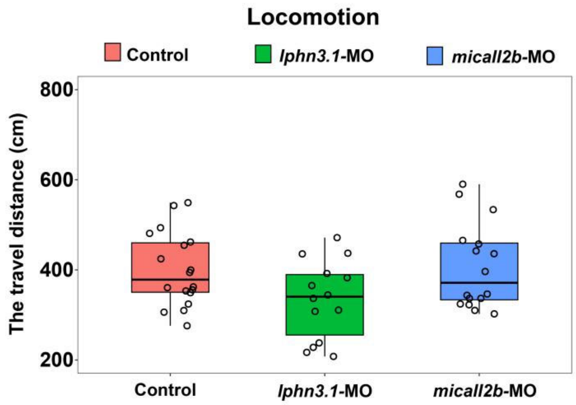

Figure S3. locomotion of 14dpf zebrafish after *lphn3.1* and *micall2b* gene knockdown. Box-and-whisker plot displaying average swimming distance (cm) across three experimental groups: control, *lphn3.1*-MO, and *micall2b*-MO zebrafish (n = 4 independent tanks per group; 3-5 larvae subsampled from each tank; One-way ANOVA with Tukey' s post-hoc test, *p*-value = 0.0834). All values are presented as the mean of tank-averaged values ± SEM. All values are presented as the mean of tank-averaged values ± SEM. Overlaid dots denote data from individual larvae.
